# The structural relationships among Korean golf instructors’ human service quality, customer emotional response, customer satisfaction, and customer learning transfer

**DOI:** 10.3389/fpsyg.2025.1663428

**Published:** 2025-10-02

**Authors:** Ki Hong Kwon, Yong-Ki Kim

**Affiliations:** ^1^School of Tourism and Hospitality Management, Sanya University, Hainan, China; ^2^Chonbuk Science College, Jeongeup, Republic of Korea

**Keywords:** golf instruction, service quality, emotional response, customer satisfaction, learning transfer, structural equation modeling

## Abstract

This study investigates the structural relationships among golf instructors’ human service quality, customers’ emotional responses, customer satisfaction, and learning transfer in the context of golf lesson participants in South Korea. The research focuses on adult golfers who received instruction within the past 2 years at outdoor golf practice ranges. Data were collected from 376 valid responses using a structured questionnaire and analyzed using structural equation modeling (SEM). The results reveal that the human service quality of golf instructors has a significant positive effect on customers’ positive emotional responses and a significant negative effect on their negative emotional responses. Furthermore, both the instructors’ service quality and customers’ positive emotional responses significantly contribute to higher customer satisfaction. Conversely, negative emotional responses were found to decrease satisfaction. Regarding learning transfer, customer satisfaction positively influences the extent to which golf lesson content is effectively applied, while negative emotional responses negatively affect this process. Mediation analysis further indicates that the impact of human service quality on learning transfer is significantly mediated by both customer satisfaction and emotional responses. In particular, positive emotional responses enhance learning transfer through increased satisfaction, suggesting a dual pathway of influence. These findings underscore the importance of emotional experience and perceived service quality in sports instruction settings. Golf instructors should prioritize strategies that foster positive emotional experiences and satisfaction to optimize learning outcomes and promote the transfer of skills from training to actual performance.

## 1 Introduction

In recent years, interest in golf has been rapidly increasing within Korean society. This phenomenon can be largely attributed to the widespread dissemination of golf-related content across various media, including entertainment programs, YouTube, and diverse online video platforms. Such media exposure has not only enhanced public awareness and understanding of golf but has also played a significant role in promoting both interest in and participation in the sport. As a result, the golfing population has continued to grow, with a notable trend among beginners seeking to acquire foundational skills and knowledge at practice ranges prior to gaining actual field experience (Youn, 2021).

Effective golf learning requires systematic professional instruction, underscoring the critical role of “lesson pros”—instructors operating at facilities equipped with appropriate practice infrastructure. Lesson pros provide comprehensive guidance that covers basic swing techniques, on-course etiquette, methods for controlling ball trajectory, strategies for managing stress during play, and effective game management. Moreover, by conducting on-course lessons, lesson pros assess how well learners apply the acquired skills in real game situations, thereby making a substantial contribution to the improvement of actual playing performance.

Within this context, the concept of learning transfer serves as a key evaluative criterion, referring to the extent to which knowledge or skills acquired through lessons are effectively manifested on the golf course (Kwak, 2011). Learning transfer generally denotes the sustained and effective application of competencies gained through training in real-world environments (Lee, 2009), and in golf, it specifically refers to the ability to execute techniques and movements learned from lesson pros during actual play (Kwak, 2017).

Previous studies have primarily focused on the relationship between golf instructors’ leadership and skill acquisition (Kim, 2013; Shin and Lee, 2017) or on how teaching presence, instructional style, and interpersonal characteristics—such as kindness, attitude, and communication ability—influence learning efficiency and outcomes (Kong et al., 2017; Lee and Kim, 2015). These diverse instructor-related attributes are collectively conceptualized as human service quality, which has been identified as a critical factor influencing learners’ service experiences, overall satisfaction, and tangible learning outcomes. For example, Moon (2018) and Ji (2018) reported that golf instructors contribute to the enhancement of both technical and physical performance, thereby increasing customer satisfaction, while Lee et al. (2018) demonstrated that human service quality has a significant positive effect on customers’ positive emotions. Conversely, Kwak (2011) indicated that a lack of service or unfriendliness can induce negative emotional reactions.

Synthesizing these findings, it can be concluded that human service quality primarily influences two psychological variables: customer emotional reactions and customer satisfaction. Emotional reactions are internal psychological states triggered by specific situations or stimuli, encompassing a range of experiences such as anxiety, joy, fatigue, or anger (Kwon, 2005). Customer satisfaction is defined as a positive evaluation that occurs when the quality of service or facilities meets or exceeds customer expectations (Lee, 2007).

Importantly, prior research has highlighted the mediating roles of emotional reactions and satisfaction in the relationship between service quality, behavioral intention, and participation continuity. For instance, Joo et al. (2013) found that emotional intelligence and non-verbal communication influence the intention to continue participation indirectly via exercise immersion and customer satisfaction. Similarly, Choi and Cheon (2012) reported that the attractiveness of human resources at sports centers affects behavioral intentions through users’ negative emotional reactions.

However, previous research has not clearly identified the structural mechanisms through which human service quality ultimately leads to actual learning transfer in golf. Most prior studies have either focused on intermediate variables such as behavioral intention or user satisfaction, or treated emotional responses and satisfaction as separate factors rather than sequential mediators. Furthermore, few empirical models have been tested that integrate emotional reactions and satisfaction as dual mediating pathways in the context of golf instruction. This gap limits the practical applicability of existing findings, particularly in designing instructional strategies that enhance the transfer of learning to real-game performance.

Accordingly, the present study aims to systematically examine the structural relationships among human service quality of golf instructors, customer emotional reactions, customer satisfaction, and learning transfer, specifically targeting Korean golf participants. By integrating psychological and instructional perspectives, this research tests a dual mediation model where emotional reactions and satisfaction sequentially mediate the effect of human service quality on learning transfer. The study seeks to provide a more comprehensive understanding of how service-related instructional factors translate into real-world golf performance, thereby offering both theoretical and practical contributions to the sport pedagogy and golf industry.

## 2 Research methods

### 2.1 Research subjects

The participants in this study consisted of adult men and women recruited from five outdoor golf driving ranges located in Seoul, South Korea. Each facility included more than 50 hitting bays, ensuring a high volume of visitors and regular instructional activity. A total of 400 individuals who had received golf lessons from certified instructors within the past 2 years were initially surveyed using a structured questionnaire.

To ensure the integrity of the data, responses were screened for completeness and consistency. After excluding 24 questionnaires due to insincere or incomplete responses, a final sample of 376 participants was retained for analysis. The demographic characteristics of the participants, including age, gender, education level, and golfing experience, are presented in Table 1.

**Table 1:** Demographic features of subject of study.

Section		Frequency	%
Age	Male	207	55.1
	Female	169	44.9
	20’s	60	16.0
	30’s	138	36.7
	40’s	129	34.3
	50’s	43	11.4
	60’s and over	6	1.6
Career	Under 1y		20.2
	Under 1 ~ 3y		32.7
	Under 3 ~ 5y		33.0
	Under 5 ~ 10y		9.6
	Over 10y		4.5
Total		376	100%

All participants were informed of the purpose and voluntary nature of the study, and written informed consent was obtained prior to participation. Ethical standards in accordance with the Declaration of Helsinki were followed throughout the data collection process.

### 2.2 Research hypothesis

**Figure 1:**
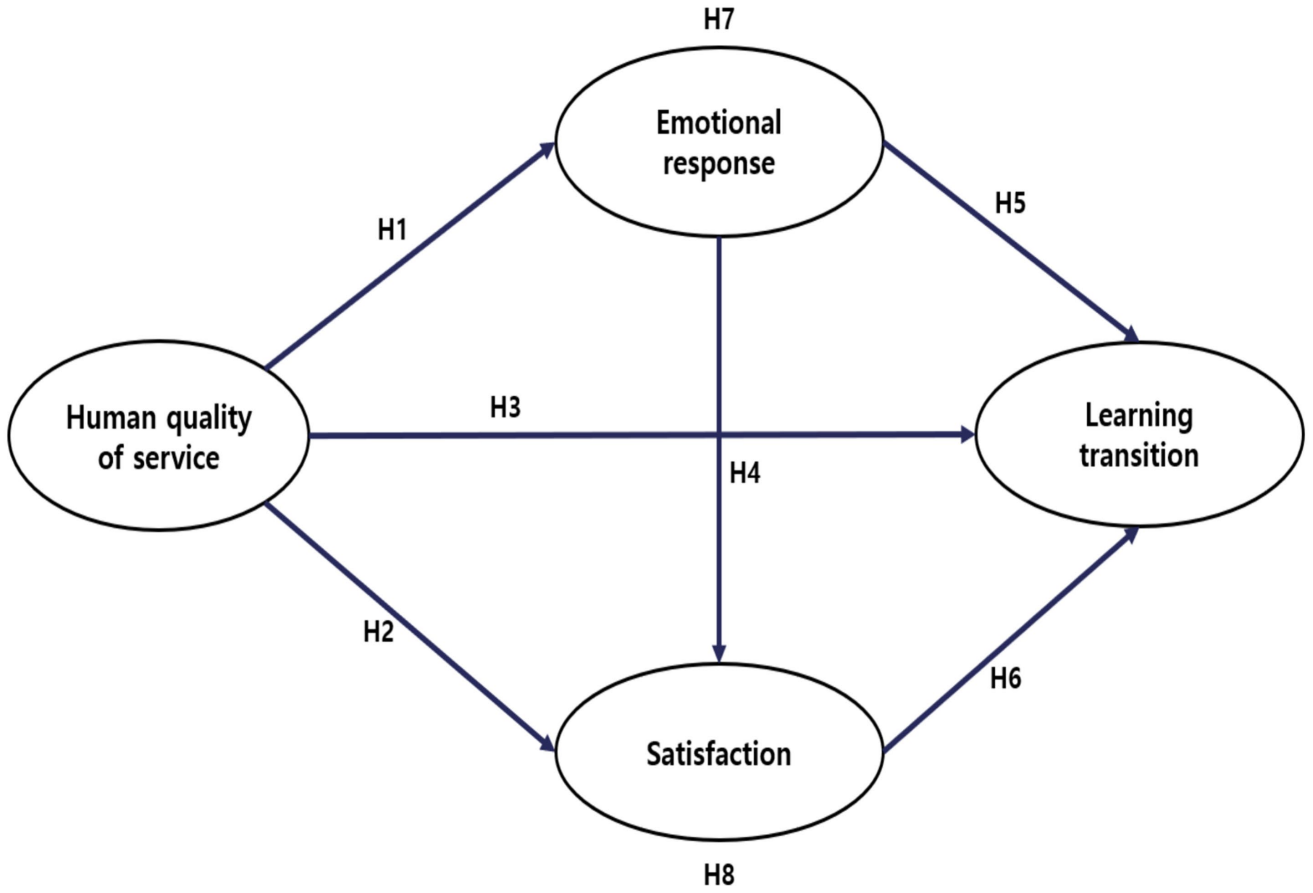
Hypothctical rcscarch modcl.

Drawing on prior research in service quality, emotional psychology, customer satisfaction, and educational transfer theory, this study formulated the following hypotheses to explore the structural relationships among the key constructs. These hypotheses were tested using the structural model presented in Figure 1.

#### 2.2.1 Direct effects

*H1*: The human service quality of golf instructors has a significant effect on customers’ emotional responses.

*H2*: The human service quality of golf instructors has a significant effect on customer satisfaction.

*H3*: The human service quality of golf instructors has a significant effect on customer learning transfer.

*H4:* Customers’ emotional responses have a significant effect on customer satisfaction.

This directional assumption is grounded in *affect-as-information theory*, which suggests that individuals use emotional states as heuristic cues when forming evaluative judgments such as satisfaction. In emotionally engaging environments such as golf instruction, positive emotional experiences during lessons are likely to shape learners’ perceptions of satisfaction. In contrast, the reverse direction—satisfaction influencing emotions—has been less supported in affective decision-making literature, particularly in instructional settings where emotions are immediate and formative.

*H5*: Customers’ emotional responses have a significant effect on customer learning transfer.

*H6*: Customer satisfaction has a significant effect on customer learning transfer.

#### 2.2.2 Mediated effects

*H7*: The effect of golf instructors’ human service quality on customer learning transfer is mediated by customers’ emotional responses.

*H8*: The effect of golf instructors’ human service quality on customer learning transfer is mediated by customer satisfaction.

These hypotheses aim to capture both the direct and indirect mechanisms through which human service quality contributes to learning effectiveness in the context of golf instruction. By examining emotional and cognitive mediators, the model seeks to provide a comprehensive understanding of how instructional service quality translates into actual learning outcomes.

### 2.3 Survey tool

The survey tool, in the form of a questionnaire, designed to identify the relationships between the quality of personal service provided by golf instructors and customer emotional responses, customer satisfaction, and customer learning transfer is shown in Table 2.

**Table 2:** Questionnaire configuration.

Category	Content composition	Question
Demographic characteristics	Gender (1), age (1), academic background (1), job (1), income (1), career (1), number of lessons (1)	7
Human quality of service	Tangibility (5), reliability (4), empathy (5)	14
Emotional response	Positive emotional response (5), negative emotional response (5)	10
Satisfaction	Satisfaction (4)	4
Learning transition	learning transition (6)	6
Total		41

#### 2.3.1 All items were rated using a 5-point Likert scale

First, the questionnaire on the quality of personal service provided by golf instructors was based on the surveys by Kim (2015) and Lee et al. (2018) with modifications and additions to fit the context of this study.

Secondly, the customer emotional response scale was derived from the surveys used by Cho (2015) and Kung et al. (2016) with modifications and additions to suit the context of this study.

Thirdly, the customer satisfaction scale was derived from the surveys used by Ha (2012) and Seol and Kim (2010) with modifications and adjustments to fit the context of this study.

Fourthly, the customer learning transfer scale was derived from the study by Rouiller and Goldstein (1993) with modifications and adjustments made by Kwak (2017) to suit the context of this study.

### 2.4 Validation of the questionnaire

To validate the questionnaire used in this study, confirmatory factor analysis (CFA) was performed using AMOS 24.0 to ensure the unidimensionality of the observed variables for the latent constructs. After validating the questionnaire, Cronbach’s *α* coefficient was used to verify the reliability of the measurement tool.

First, the results of the confirmatory factor analysis (CFA) for the personal service quality of golf instructors are shown in Table 3. A model is considered to be a good fit if the NFI, CFI, and TLI values are 0.9 or higher, with 1 being ideal. An RMR value of 0.05 or less, and the closer to 0 the better, indicates a suitable model. An RMSEA value less than 0.08 also suggests that the model is a good fit.

**Table 3:** Confirmatory factor analysis model fit.

EI	$x^{2}$ /*df*	Q	NFI	CFI	TLI	RMR	RMSEA
HU	192.217/74	2.598	0.944	0.964	0.956	0.023	0.065
OV	678.389/309	2.195	0.923	0.957	0.951	0.026	0.056

EI, Evaluation Index; HU, Human quality of service; OV, Overall mode fit.

Secondly, the fit indices for the measurement model, including NFI, CFI, TLI, RMR, and RMSEA, met the criteria for determining model fit. Therefore, a convergent validity analysis was conducted on the measurement model. Convergent validity is achieved when there is a high correlation between measurement values in a given set of items designed to measure the same concept established by the researcher.

It assesses whether the observed variables represent the latent variable well. The presented standardized coefficients are all above 0.50, indicating statistical significance. The average variance extracted (AVE) exceeds 0.50, and the composite reliability (CR) is above 0.70, meeting the criteria. These results demonstrate that convergent validity has been achieved.

The standardized coefficients, standard errors, critical ratios (CR), average variance extracted (AVE), and composite reliability (CR) were calculated for each path, with the standardized coefficients serving the same purpose as factor loadings in confirmatory factor analysis. In this study, these values were presented in Table 4.

**Table 4:** Confirmatory factor analysis results for the measurement model.

	*B*	*β*	*S.E.*	C.R	AVE	CR
EM ← HU	1.000	0.898			0.891	0.961
RE ← HU	0.932	0.890	0.036	26.228^***^		
TA ← HU	0.973	0.888	0.037	26.085^***^		
PO3 ← PO	1.000	0.731				
PO2 ← PO	1.155	0.795	0.075	15.297^***^		
PO1 ← PO	0.938	0.699	0.070	13.367^***^	0.656	0.905
PO4 ← PO	1.030	0.744	0.072	14.262^***^		
PO5 ← PO	1.107	0.790	0.073	15.191^***^		
NE8 ← NE	1.000	0.890				
NE7 ← NE	1.014	0.869	0.042	24.155^***^		
NE6 ← NE	0.990	0.826	0.046	21.751^***^	0.717	0.927
NE9 ← NE	0.978	0.893	0.038	25.645^***^		
NE10 ← NE	1.018	0.892	0.040	25.565^***^		
SA2 ← SP	1.000	0.771				
SA1 ← SP	0.911	0.744	0.060	15.166^***^		
SA3 ← SP	0.909	0.681	0.066	13.675^***^	0.661	0.886
SA4 ← SP	0.986	0.780	0.061	16.033^***^		
LT3 ← LT	1.000	0.758				
LT2 ← LT	0.980	0.734	0.068	14.446^***^		
LT1 ← LT	0.842	0.683	0.063	13.332^***^		
LT4 ← LT	1.035	0.774	0.067	15.343^***^	0.674	0.925
LT5 ← LT	0.978	0.770	0.064	15.260^***^		
LT6 ← LT	0.920	0.736	0.064	14.488^***^		

HU, Human quality of service; TA, Tangibility; RE, Reliability; EM, Empathy; ER, Emotional response; PO, Positive emotional response; NE, Negative emotional response; SA, Satisfaction; LT, Learning transition. ***p < 0.001 indicates the conventional statistical significance level.

Discriminant validity refers to the concept that there should be a discernible difference between different latent variables, indicating that they are measuring distinct constructs.

The method for demonstrating discriminant validity involved the use of correlation coefficients and the average variance extracted (AVE). If the average variance extracted (AVE) for each construct is greater than the squared value of the correlation coefficient between latent variables, discriminant validity is considered to be established (Fornell and Larcker, 1981). However, for the cases of human service quality and positive emotional response, the correlation coefficient was found to be higher than the square root of the AVE, requiring additional verification for discriminant validity. The additional verification for discriminant validity used the standard error estimation interval for validation, resulting in the correlation ±(2*standard error) interval not including the value of 1, suggesting that discriminant validity was established. The correlations between constructs and the discriminant validity are presented in Tables 5 and 6.

**Table 5:** Correlation and discriminative validity between compositional concepts.

	HU	SE	PO	NE	SA	LT	AVE
HU	**0.944**						0.891
PO	0.811	0.829	**0.810**				0.656
NE	−0.506	−0.459	−0.483	**0.847**			0.717
SA	0.809	0.800	0.773	−0.433	**0.813**		0.661
LT	0.753	0.772	0.711	−0.462	0.768	**0.821**	0.674

The bold number on the diagonal is the square root of the AVE value, and the correlation is to compare with. HU, Human quality of service; PO, Positive emotional response; NE, Negative emotional response; SA, Satisfaction; LT, Learning transition.

**Table 6:** Discriminant validity analysis result using standard error estimation interval.

	CC	SE	*r* ± 2SE		
	*r*	SE	*r* − 2SE	*r* + 2SE	Inclusion
HU-PO	0.811	0.033	0.745	0.877	Not included

CC, Correlation coefficient; SE, standard error; HU, Human quality of service; PO, Positive emotional response.

Third, the questionnaires collected through the reliability survey were subjected to reliability verification for each sub-factor, with the results shown in Table 7. As a result, the Cronbach’s *α* coefficient was found to range from 0.810 to 0.940 for all factors. When considering a Cronbach’s *α* coefficient of 0.60 or higher as indicative of reliability, all factors showed that they met the reliability criteria.

**Table 7:** Confidence verification results.

	Factor	Cronbach’s *α*
HU	TA (5)	0.859
	RE (4)	0.810
	EM (5)	0.871
ER	PO (5)	0.851
	NE (5)	0.940
SA (4)		0.811
LT (6)		0.866

HU, Human quality of service; TA, Tangibility; RE, Reliability; EM, empathy; ER, Emotional response; PO, Positive emotional response; NE, Negative emotional response; SA, Satisfaction; LT, Learning transition.

### 2.5 Data processing

The analysis in this study was conducted using the statistical software programs SPSS 26.0 and AMOS 24.0. First, confirmatory factor analysis was carried out to validate the construct validity of the measurement tool, and Cronbach’s *α* was used to verify internal consistency. Second, frequency analysis was conducted to examine the general characteristics of the study participants, and Pearson’s product-moment correlation analysis was conducted to determine descriptive statistics, the normality of the distribution, and the relationships among variables.

Third, model fit and path analysis were conducted to test the research hypotheses, and the significance of indirect mediation effects was analyzed using the Bootstrapping technique. All statistical significance levels (*α*) were set at 0.05.

## 3 Research results

### 3.1 Descriptive statistics

Skewness with an absolute value of 3.0 or lower, and kurtosis with an absolute value of 8.0 or lower, can be used as criteria for assessing the normality of data (Rosenberg, 2009). Upon checking the skewness and kurtosis values for the measurement variables in this study, it was found that they all met these criteria. This is shown in Table 8.

**Table 8:** Descriptive statistical analysis.

	N	Mean	SD	Skewness	Kurtosis
TA	376	3.94	0.70	−0.612	0.432
RE	376	4.02	0.67	−0.493	−0.288
EM	376	3.89	0.71	−0.684	0.444
PO	376	3.91	0.67	−0.518	0.068
NE	376	2.38	1.02	0.221	−1.151
SA	376	3.93	0.65	−0.414	0.366
LT	376	3.95	0.61	−0.422	0.160

HU, Human quality of service; TA, Tangibility; RE, Reliability; EM, Empathy; ER, Emotional response; PO, Positive emotional response; NE, Negative emotional response; SA, Satisfaction; LT, Learning transition.

### 3.2 Correlation

As a result of conducting a correlation analysis on the human service quality of golf instructors in relation to customer emotional response, customer satisfaction, and customer learning transfer, Tangibility-Positive Emotional Response (*r* = 0.779, *p* < 0.001), Tangibility-Negative Emotional Response (*r* = −0.516, *p* < 0.001), Tangibility-Satisfaction (*r* = 0.760, *p* < 0.001), Tangibility-Learning Transfer (*r* = 0.695, *p* < 0.001), Reliability-Positive Emotional Response (*r* = 0.714, *p* < 0.001), Reliability-Satisfaction (*r* = 0.728, *p* < 0.001), Reliability-Learning Transfer (*r* = 0.736, *p* < 0.001), Empathy-Positive Emotional Response (*r* = 0.755, *p* < 0.001), Empathy-Satisfaction (*r* = 0.756, *p* < 0.001), Empathy-Learning Transfer (*r* = 0.682, *p* < 0.001), Positive Emotional Response-Satisfaction (*r* = 0.770, *p* < 0.001), Positive Emotional Response-Learning Transfer (*r* = 0.721, *p* < 0.001), Satisfaction-Learning Transfer (*r* = 0.778, *p* < 0.001) all showed positive correlations. Reliability-Negative Emotional Response (*r* = −0.523, *p* < 0.001), Empathy-Negative Emotional Response (*r* = −0.457, *p* < 0.001), Negative Emotional Response-Satisfaction (*r* = −0.470, *p* < 0.001), and Negative Emotional Response-Learning Transfer (*r* = −0.493, *p* < 0.001) showed negative correlations. The correlation coefficients derived from the correlation analysis between the human service quality of golf instructors and customer emotional response, customer satisfaction, and customer learning transfer are shown in Table 9.

**Table 9:** The correlation between human quality of service, emotional response, satisfaction and learning transition of golf instructors.

	TA	RE	EM	PO	NE	SA	LT
TA	1						
RE	0.784***	1					
EM	0.790***	0.799***	1				
PO	0.779***	0.714***	0.755***	1			
NE	−0.516***	−0.523***	−0.457***	−0.521***	1		
SA	0.760***	0.728***	0.756***	0.770***	−0.470***	1	
LT	0.695***	0.736***	0.682***	0.721***	−0.493***	0.778***	1

***p < 0.001, TA, Tangibility; RE, Reliability; EM, Empathy; PO, Positive emotional response; NE, Negative emotional response; SA, Satisfaction; LT, Learning transition.

### 3.3 Verification of mediation effects

In the model established for this study, a simple mediation effect analysis was conducted to examine the mediation effects of customer positive emotional response, customer negative emotional response, and customer satisfaction in the relationship between the human service quality of golf instructors and customer learning transfer. The analysis used the PROCESS Macro for SPSS (Hayes, 2018) with Bootstrapping to determine effect size and significance. The results are shown in Tables 10–12.

**Table 10:** The mediating effect of positive emotional response on the relationship between golf instructors’ human service quality and learning transfer.

Total effect	*β*	*SE*	*t*	*p*
HU → LT	0.728	0.030	24.119	***
Path	*β*	*SE*	*t*	*p*
HU → PO	0.815	0.029	28.010	***
HU → LT	0.494	0.048	10.389	***
PO → LT	0.287	0.046	6.224	0.002
Direct effect	*β*	*SE*	*t*	*p*
HU → LT	0.494	0.048	10.389	***
Indirect effect	*β*	Boot SE	Boot LLCI	Boot ULCI
HU → PO → LT	0.234	0.051	0.134	0.337

***p < 0.001, HU, Human quality of service; PO, Positive emotional response; LT, Learning transition.

**Table 11:** The mediating effect of negative emotional response on the relationship between golf instructors’ human service quality and learning transfer.

Total effect	*β*	*SE*	*t*	*p*
HU → LT	0.728	0.030	24.119	***
Path	*β*	*SE*	*t*	*p*
HU → NE	−0.867	0.064	−13.485	***
HU → LT	0.669	0.035	18.995	***
NE → LT	−0.067	0.022	−3.135	0.002
Direct effect	*β*	*SE*	*t*	*p*
HU → LT	0.669	0.035	18.995	***
Indirect effect	*β*	Boot SE	Boot LLCI	Boot ULCI
HU → NE → LT	0.058	0.021	0.018	0.102

***p < 0.001, HU, Human quality of service; NE, Negative emotional response; LT, Learning transition.

**Table 12:** The mediating effect of satisfaction on the relationship between golf instructors’ human service quality and learning transfer.

Total effect	*β*	*SE*	*t*	*p*
HU → LT	0.728	0.030	24.119	***
Path	*β*	*SE*	*t*	*p*
HU → SA	0.818	0.029	28.458	***
HU → LT	0.356	0.045	7.100	***
SA → LT	0.454	0.044	10.362	***
Direct effect	*β*	*SE*	*t*	*p*
HU → LT	0.356	0.045	7.100	***
Indirect effect	*β*	Boot SE	Boot LLCI	Boot ULCI
HU → SA → LT	0.372	0.051	0.269	0.471

***p < 0.001, HU, Human quality of service; SA, Satisfaction; LT, Learning transition.

An analysis of the mediation effect of customer positive emotional response in the relationship between the human service quality of golf instructors and customer learning transfer revealed significant total effect (*β* = 0.728, *p* < 0.001) and direct effect (*β* = 0.494, *p* < 0.001). The effect in the pathway of human service quality → positive emotional response → learning transfer (*β* = 0.234) did not contain zero within the lower and upper bounds of the 95% confidence interval, indicating statistical significance.

An analysis of the mediation effect of customer negative emotional response in the relationship between the human service quality of golf instructors and customer learning transfer found significant total effect (*β* = 0.728, *p* < 0.001) and direct effect (*β* = 0.669, *p* < 0.001). The effect in the pathway of human service quality → negative emotional response → learning transfer (*β* = 0.058) did not contain zero within the lower and upper bounds of the 95% confidence interval, indicating statistical significance.

An analysis of the mediation effect of customer satisfaction in the relationship between the human service quality of golf instructors and customer learning transfer revealed significant total effect (*β* = 0.728, *p* < 0.001) and direct effect (*β* = 0.356, *p* < 0.001). The effect in the pathway of human service quality → satisfaction → learning transfer (*β* = 0.372) did not contain zero between the lower and upper bounds within the 95% confidence interval, indicating statistical significance.

## 4 Discussion

This study uses structural equation modeling to analyze the structural relationships among the personal service quality of Korean golf instructors, customer emotional responses, customer satisfaction, and customer learning transfer, identifying the causal relationships among the sub-factors. The discussion on the results of this study is as follows.

First, an analysis of the relationship between the personal service quality of golf instructors and customer emotional responses reveals that in a study conducted by Ji (2018) with golf range customers, the tangibility, empathy, and reliability among the factors of personal service quality had a significant positive (+) effect on positive emotional responses. Moon (2018) supported the findings of this study by reporting that among the sub-factors of personal service quality, empathy and reliability had a significant positive (+) effect on positive emotional responses in a study with customers using golf courses.

Additionally, Ji (2018) support the findings of this study by reporting that among the sub-factors of personal service quality, tangibility, reliability, and empathy had a significant positive (+) effect on elements of customer emotion, such as joy, gratitude, and excitement, in a study conducted with screen golf participants.

Therefore, all sub-factors of personal service quality are antecedents that have a significant positive (+) impact on the positive emotional responses of golf customers. Accordingly, tangibility, which relates to the golf instructor’s appearance, speech, politeness, and attire, as well as reliability, which focuses on teaching methods that meet customer needs and emphasizes professional knowledge, and empathy, which shows interest in and empathy for the customer and responds promptly to customer requests, are factors that promote positive emotional responses from customers.

This can serve as a catalyst for forming positive emotional responses in customers, and it is believed that these positive emotional responses may also impact the overall perception of the golf driving range in a favorable manner.

Secondly, the analysis of the relationship between the personal service quality of Korean golf instructors and customer satisfaction shows that Moon and Hur (2016), in a study with golf driving range customers, found that the sub-factors of personal service quality, namely tangibility, reliability, and empathy, had a significant positive (+) impact on customer satisfaction, which aligns with the results of this study. Seol and Kim (2010), in a study with customers using screen golf, found that sub-factors such as interest in the customer, understanding customer needs, punctuality, and rapid problem-solving ability had a significant positive (+) impact on customer satisfaction. This supports the findings of this study.

Based on the above results, due to the unique characteristics of golf, learning involves close interaction with customers, unlike conventional lectures. Therefore, factors like tone of voice, attire, appearance, scientific approach to learning, professional knowledge, and active interest can help facilitate customers’ clear understanding during the learning process.

Therefore, while verbal communication is important in conducting learning, enhancing other aspects like environment, appearance, and instructional methods can help maintain customer focus on the instructor, which could lead to greater customer satisfaction.

Thirdly, the analysis of the relationship between the personal service quality of Korean golf instructors and learning transfer revealed that Seo et al. (2007) in a study examining golf instructors’ instructional behavior and interactions with learners, reported that when golf instructors deliver clear content knowledge and provide positive feedback such as encouragement and praise, there is a significant positive (+) impact on the learners’ ability to transfer learning. The results of this study support the conclusion that the sub-factors of personal service quality in golf instructors—specifically, tangibility, reliability, kindness, and proactive interest—have a significant positive (+) effect on learning transfer. Lee and Kim (2015), in a study with members who have taken lessons at golf driving ranges, reported that the sub-factors of golf lesson instructors’ qualifications, including scientific instruction, knowledge of lessons, and delivery skills, have a significant positive (+) impact on learning efficiency. This supports the results of the current study, indicating that the reliability sub-factor within the personal service quality of golf instructors has a significant positive (+) impact on learning transfer. No (2004) in a study with customers at golf driving ranges, reported that elements related to tangibility within personal service quality, such as appearance, attire, facial expressions, politeness, and kindness, contribute to a positive impression, leading to continued participation. This supports the results of the current study.

Based on the results above, it is advisable for golf instructors to focus on aspects related to tangibility, such as appearance, attitude, and voice. By creating an environment where customers feel positive and can concentrate on learning, and by adhering to schedules while using the necessary professional knowledge and tools to explain concepts clearly, instructors can significantly enhance on-field performance. An important factor in helping customers perform well is for instructors to identify and address what the customers need. By making an effort to understand and resolve customer requirements, instructors can provide guidance and support, enabling customers to improve their skills and confidence.

Fourthly, an analysis of the impact of customer emotional responses on customer satisfaction revealed that Kung et al. (2016), in their study on the effect of customer emotional responses on leisure satisfaction among golf driving range users, found that positive emotions had a significant positive (+) effect on leisure satisfaction. This indicates that the higher the positive emotions of customers using golf driving ranges, the more likely they are to continue enjoying golf as a leisure activity. These findings partially support the results of this study. Ha (2012) in a study with golf course users, reported that positive emotions had a significant positive (+) impact on customer satisfaction. He suggested that considerable efforts should be made and methods devised to ensure that customers develop positive emotions, highlighting the importance of fostering a positive emotional environment for improving customer satisfaction.

Based on the above, it is necessary to understand where positive emotions originate among customer emotion factors to increase customer satisfaction. This directional assumption is grounded in affect-as-information theory, which posits that individuals use their emotional states as heuristic cues when making evaluative judgments such as satisfaction (Schwarz and Clore, 1983). In the context of golf instruction, emotional experiences—such as joy, gratitude, or frustration—serve as meaningful internal signals that shape learners’ overall evaluation of the service. Empirical research in consumer behavior similarly supports this causal path, showing that affective responses experienced during service consumption consistently predict satisfaction (Erevelles, 1998). Since emotional responses tend to arise early in interactions and influence reflective evaluations later, the assumed causal path from emotional response to satisfaction is both theoretically and empirically justified in this study.

If the cause of the problem is attributed to the customer, the impact on customer satisfaction may be reduced. However, if the issue arises from the personal service provided, it is crucial to address it promptly from the participant’s perspective to prevent the development of negative emotions. This approach can help ensure that negative emotions do not arise due to service-related issues.

Fifth, an analysis of the impact of customer emotional responses on customer learning transfer showed that Kwak (2011) in a study with golf driving range customers, reported that positive emotional responses from customers had a significant positive (+) impact on the sub-factors of attitude, particularly in terms of enhanced liking and closeness. He also noted that negative emotions were partially accepted, supporting the findings of this study. No and Oh (2014) in a study with golf driving range users, reported that sub-factors of customer emotional responses, such as joy and arousal, had a significant positive (+) impact on the sub-factor of sustained use under continued behavior. Kim (2010) in a study with golf driving range customers, reported that the sub-factor of joy in emotional responses had a significant positive (+) impact on exercise continuation intention, supporting the findings of this study.

Based on the above, the positive emotional sub-factors, such as comfort, cheerfulness, contentment, and happiness, contrast with the negative emotional sub-factors, like annoyance, unpleasantness, anger, regret, and disappointment, all of which influence learning transfer. This implies that positive emotions are likely to promote learning transfer more effectively than negative emotions. Therefore, to enhance learning transfer, fostering positive emotions in customers could be more beneficial than addressing negative emotions.

Sixth, the analysis of the relationship between customer satisfaction and customer learning transfer showed that Won et al. (2019) in a study with golf driving range customers, reported that customer satisfaction had a significant positive (+) impact on continuance intention. This finding supports the results of the current study. Lee (2007) in a study with middle and high school golf players, reported that task performance satisfaction and instructor behavior satisfaction had a significant positive (+) impact on cognitive performance, supporting the results of the current study. Additionally, Jo and Moon (2011) in a study with golf players, reported that environmental satisfaction and athlete life satisfaction had a significant positive (+) impact on performance, supporting the results of the current study.

Based on the above, when golf customers experience comprehensive satisfaction with the instructor’s expression, attitude, and the professional knowledge and skills needed for learning, they are more likely to focus and train more effectively. It is believed that if customers apply what they have learned in training to the field, it will help improve their skills and performance.

Seventh, it was confirmed that the personal service quality of Korean golf instructors not only directly impacts learning transfer but also influences customer learning transfer through the mediation of customer positive emotional responses.

These results align with the findings of Han and Kang (2015) which showed that the personal service provided by sports centers has a significant impact on customer behavior through the mediation of customer positive emotions. Additionally, this finding aligns with the research results of Park and Kwon (2016) indicating that the non-verbal communication of fitness instructors has a significant impact on customer loyalty through the mediation of positive customer responses. Since the personal service quality of golf instructors has a direct impact on learning transfer and also influences it through positive emotional responses, efforts to improve service quality, along with strategies to induce positive emotional responses in learning customers, are needed to strengthen the effect of learning transfer. The mediating effect of negative emotional responses in the relationship between the personal service quality of golf instructors and learning transfer was found to be statistically significant, indicating a partial mediating role. It was confirmed that the personal service quality of golf instructors directly affects learning transfer, and also influences learning transfer through the mediation of negative emotional responses in learning customers.

These results are consistent with the findings of Choi and Cheon (2012) which showed that the attractiveness of human resources in sports centers has a significant impact on customer behavior through the mediation of negative emotional responses. Therefore, as the personal service quality of golf instructors has a direct impact on learning transfer and also affects it through negative emotional responses, it is crucial to minimize negative emotions by addressing customer complaints and demands to enhance the effectiveness of learning transfer.

Eighth, it was confirmed that customer satisfaction, set as a mediating variable, showed statistical significance in the relationship between the human service quality of Korean golf instructors and the dependent variable of learning transfer. This result aligns with the study by Jung and Lee (2020) which showed that the human service quality of adult ballet instructors has a significant impact on customers’ future behavioral intentions through mediation by customer satisfaction.

Based on the above content, the quality of human services provided by golf instructors directly influences the learning transfer of students, while also affecting it through customer satisfaction. Therefore, to enhance the effect of learning transfer, golf instructors should always pay attention to any areas where customers might feel dissatisfied and actively monitor those aspects.

## 5 Conclusion and implications

The primary aim of this study was to examine the structural relationships between the human service quality of Korean golf instructors and customers’ emotional responses (both positive and negative), satisfaction, and learning transfer. Based on the structural equation modeling results, several meaningful implications can be discussed.

First, the findings revealed that human service quality was positively associated with learners’ positive emotional responses and negatively associated with negative emotional responses. These emotional reactions, in turn, showed significant associations with satisfaction and learning transfer. Specifically, positive emotional responses were positively related to satisfaction, which was then positively associated with learning transfer. Conversely, negative emotional responses were negatively related to learning transfer. These associations suggest that emotional and cognitive reactions function as key psychological pathways through which perceived instructional quality may relate to skill application outcomes in real golf settings.

Second, the mediating roles of emotional responses and satisfaction were confirmed. Satisfaction significantly mediated the relationship between human service quality and learning transfer (indirect effect: *β* = 0.162), while negative emotional responses also mediated this path (*β* = −0.084). Additionally, positive emotional responses had an indirect influence on learning transfer via satisfaction (*β* = 0.126). The total mediation proportion accounted for approximately 41.2% of the total effect from human service quality to learning transfer. However, it should be noted that the variance explained (*R*^2^) by personality traits and emotional responses was modest. This suggests that other unmeasured variables may also contribute to learning transfer, warranting future investigation. These results provide empirical support for the dual-pathway mediation model and emphasize the importance of both affective and evaluative processes in explaining instructional effectiveness (Hayes, 2018).

From a practical standpoint, the findings highlight the importance of creating emotionally supportive and learner-centered instructional environments. Golf instructors are encouraged to improve not only their technical instruction but also their relational and emotional service capacities—such as empathy, active listening, and emotional responsiveness—which can increase satisfaction and promote effective learning transfer. Instructor training programs should incorporate service quality dimensions to foster emotional engagement and trust with learners.

Academically, this study contributes to the literature by integrating human service quality, emotion, and transfer into a single structural framework within the context of individual sport instruction. It extends service quality theory by empirically testing psychological mechanisms that potentially link instructional experiences to behavioral outcomes in physical activity settings.

To avoid overstatement, it is important to note that while the results are consistent with the proposed causal directions, the cross-sectional nature of the study limits the ability to claim causality. The relationships identified should be interpreted as statistically significant associations rather than definitive cause-and-effect conclusions.

## 6 Limitations and future research directions

### 6.1 Methodological limitations

First, although confirmatory factor analysis (CFA), reliability, and discriminant validity assessments were conducted, the high correlation between human service quality and positive emotional response suggests a potential issue with discriminant validity. Future studies should consider applying more rigorous validation methods such as the heterotrait–monotrait (HTMT) ratio to further assess construct distinctiveness.

Second, this study employed a cross-sectional research design, which restricts the ability to infer temporal or causal relationships among variables. While structural equation modeling can suggest plausible paths, longitudinal or experimental studies are needed to establish causality with greater confidence.

Third, model fit indices for the final SEM model were not fully reported. To enhance replicability and transparency, future research should provide a dedicated table presenting fit statistics, including *χ*^2^, CFI, TLI, RMSEA, and SRMR. In addition, future studies should report effect sizes and mediation proportions to enhance the interpretability of indirect effects in mediation models (Hayes, 2018). Additionally, the explanatory power (*R*^2^) for some endogenous variables was modest, suggesting that unmeasured variables may influence the outcomes. Future studies should incorporate additional predictors to improve model comprehensiveness.

### 6.2 Sample characteristics and generalizability

The generalizability of the findings is limited by the use of a geographically restricted sample—participants were recruited from five golf driving ranges in the Seoul metropolitan area. Future studies should consider more diverse samples, including learners from other regions or countries, to examine potential cultural and contextual differences.

Furthermore, all participants were adult learners who had taken lessons within the past 2 years. Although this criterion ensured data relevance, the potential for recall bias should be acknowledged. Real-time data collection methods, such as post-lesson experience sampling, could mitigate such limitations.

### 6.3 Contextual and instructor-related limitations

Several contextual and methodological limitations remain unaddressed. For instance, the study did not control for contextual factors such as facility conditions, weather, or learners’ initial motivation—variables that could plausibly influence emotional responses, satisfaction, or learning transfer outcomes. Additionally, personality traits were measured using a brief instrument (e.g., TIPI), which may limit the depth and nuance of personality assessment. While the brevity offers practicality, it may not fully capture the complexity of individual differences. Future studies should consider employing more comprehensive personality inventories to enhance measurement validity.

Similarly, variation in instructor teaching styles was not systematically accounted for, even though pedagogical approaches may differentially affect learners’ emotional and cognitive engagement. Future research should include these contextual and instructor-level variables to enhance model robustness and ecological validity.

### 6.4 Self-report bias

The reliance on self-reported data introduces the possibility of social desirability bias, where respondents may overstate positive emotions or learning gains to conform to perceived expectations. Triangulating self-reports with observational data or instructor assessments could strengthen data validity.

### 6.5 Analytical recommendations

Future research should expand the analytical approach by reporting standardized indirect effects and mediation proportions to improve interpretability and application. Such analyses could help quantify the extent to which instructional quality is transmitted through affective and cognitive processes, ultimately informing effective teaching practices in sport contexts.

To address these limitations and extend the current findings, future studies are encouraged to employ multi-group analyses to examine whether the proposed structural relationships hold consistently across learner subgroups (e.g., by age, gender, or golf experience level). Furthermore, alternative model structures—such as reversing the sequence from satisfaction to emotion to learning transfer—could be tested using nested model comparisons to verify the robustness and directionality of the assumed causal paths.

### 6.6 Global implications

While this study focused on golf instruction within the Korean context, its practical implications could be expanded to the broader global golf industry. Exploring cross-cultural differences in service expectations, emotional dynamics, and instructional outcomes would offer more comprehensive insights for international applications.

## Data Availability

The raw data supporting the conclusions of this article will be made available by the authors, without undue reservation.

## References

[ref1] Cho N. H. (2015). The effects of leisure dance instructor's personal service on learner’s emotional reaction and customer loyalty. Korean Soc. Wellness 10, 197–212. Available at: https://www.kci.go.kr/kciportal/ci/sereArticleSearch/ciSereArtiView.kci?sereArticleSearchBean.artiId=ART002051462

[ref2] Choi M. S. Cheon W. K. (2012). An effect of emotional reaction by human service and attractiveness of sports center staffs upon behavioral intention. Korean J. Sports Sci. 21, 413–424. Available at: https://www.kci.go.kr/kciportal/ci/sereArticleSearch/ciSereArtiView.kci?sereArticleSearchBean.artiId=ART001709766

[ref3] Erevelles S. (1998). The role of affect in marketing. J. Bus. Res. 42, 199–215. doi: 10.1016/S0148-2963(97)00102-4

[ref4] Fornell C. Larcker D. F. (1981). Evaluating structural equation models with unobservable variables and measurement error. J. Mark. Res. 18, 39–50. doi: 10.2307/3151312

[ref5] Ha S. W. (2012). The relationship among service quality, service value, cognitive and affective responses, customer satisfaction, revisit intentions, and loyalty of golf courses. (doctoral dissertation). Dankook University

[ref6] Han Y. J. Kang H. W. (2015). The relationship between servicescape and human service and customer emotional response and behavioral intentions in commercial sports centers. Korean J. Soc. Sports 59, 235–247. doi: 10.51979/KSSLS.2015.02.59.235

[ref7] Hayes A. F. (2018). Introduction to mediation, moderation, and conditional process analysis: a regression-based approach. 2nd Edn. New York, NY: The Guilford Press.

[ref8] Ji J. C. (2018). The relationship among personal service quality, participants’ emotional response, and lesson satisfaction of golf instructors. Korean J. Sport 16, 881–891. Available at: https://www.earticle.net/Article/A344188

[ref9] Jo M. Moon J. Y. (2011). The causal relationship between self-management, exercise satisfaction, and performance among KLPGA players depending on their participation tour. Korean J. Sport Sci. 20, 647–660. Available at: https://www.kci.go.kr/kciportal/ci/sereArticleSearch/ciSereArtiView.kci?sereArticleSearchBean.artiId=ART001585993

[ref10] Joo H. C. Yoon I. A. Kim J. Y. (2013). The effect of instructors’ emotional intelligence and nonverbal communication on exercise commitment, customer satisfaction, and intention of participation continuity as perceived by GX program participants. Korean J. Phys. Educ. 52, 267–284. Avaiable at: https://www.kci.go.kr/kciportal/ci/sereArticleSearch/ciSereArtiView.kci?sereArticleSearchBean.artiId=ART001825337

[ref11] Jung T. G. Lee Y. J. (2020). The relationship between the personal service quality of adult ballet instructors, service value, satisfaction, and future behavioral intentions. Korean J. Sports Sci. 29, 317–330. doi: 10.35159/kjss.2020.02.29.1.317

[ref12] Kim H. S. (2010). The causal relationship between service quality, customer satisfaction, continuance intention, and recommendation intention at golf practice ranges. Korean J. Soc. Phys. Educ. 41, 403–417. doi: 10.51979/KSSLS.2010.08.41.403

[ref13] Kim Y. R. The effect of feedback precision on transfer of golf putting learning Korea Sport Soc (2013) 11 293–301. Available online at: https://www.earticle.net/Article/A200500

[ref14] Kim M. S. (2015) An effect of sports center human service quality and service recovery on customer loyalty. (doctoral dissertation). Pusan National University

[ref16] Kong J. Y. Lee H. G. Shin S. M. (2017). The causal model of perceived teaching presence, learning presence and learning effects of TV golf lesson program. Korean Soc. Stud. Phys. Educ. 22, 109–122. doi: 10.15831/JKSSPE.2017.22.2.109

[ref17] Kung S. T. Jung C. K. Kim H. R. (2016). The effect of service scape of a golf course on customer feeling response and leisure satisfaction. Korean J. Sport 14, 147–159. Available at: https://www.kci.go.kr/kciportal/ci/sereArticleSearch/ciSereArtiView.kci?sereArticleSearchBean.artiId=ART002188169

[ref18] Kwak H. P. (2011). The effect of service quality on emotional response and attitude to the service interface of a golf range. Korean J. Sports Sci. 20, 31–44. Available at: https://www.dbpia.co.kr/journal/articleDetail?nodeId=NODE01637471

[ref19] Kwak J. W. (2017). The effects of golf coach’s leadership type on exercise commitment and learning transition. (doctoral dissertation). Hanyang University

[ref20] Kwon S. T. (2005). The research of affected action and mental reaction of consumer from restaurant interior design. (doctoral dissertation). Kyonggi University

[ref21] Lee S. E. (2007). The effects of leadership styles of golf instructors on player satisfaction and performance. (doctoral dissertation). Kyonggi University

[ref22] Lee J. S. (2009). A study on a structural equation model of the effects of learners' personal characteristics and organizational transfer support on near- and far-transfer of learning: A case of public officials training. (master’s thesis). Korea University, Sejong

[ref23] Lee Y. G. Joo H. C. Lee M. S. (2018). The relationship between human service quality and customer emotion, trust, and future behavioral intentions at screen golf facilities. Korean J. Sports Sci. 27, 515–531. doi: 10.35159/kjss.2018.02.27.1.515

[ref24] Lee D. H. Kim B. S. (2015). The effect of teaching method type and quality of golf lesson pro on learning efficiency and continuous participation intention in golf practice range. J. Sport Leis. Stud. 61, 405–420. doi: 10.51979/KSSLS.2015.08.61.405

[ref25] Moon C. W. (2018). The relationship between human service quality and customer’s emotional response of coaches in golf. (master’s thesis). Korea National Sport University. Available at: https://www.riss.kr/search/detail/DetailView.do?p_mat_type=be54d9b8bc7cdb09&control_no=cb2baba3b458a0e0ffe0bdc3ef48d419&keyword

[ref26] Moon S. H. Hur J. (2016). The effects of servicescape and human service on customer satisfaction, switching cost, and loyalty in golf practice ranges. Korean J. Golf Stud. 10, 81–90.

[ref27] No D. Y. (2004). The relationship between perceived service quality, service value, customer satisfaction, and intention to reuse among consumers of sports centers. Korean J. Sport Sci. 9, 71–88. Available at: https://www.kci.go.kr/kciportal/ci/sereArticleSearch/ciSereArtiView.kci?sereArticleSearchBean.artiId=ART001165001

[ref28] No D. Y. Oh Y. S. (2014). The effect of servicescape at golf practice ranges on customer emotional response and continuing behavior. Korean J. Sports Sci. 23, 815–826. Available at: https://www.dbpia.co.kr/Journal/articleDetail?nodeId=NODE02440568

[ref29] Park J. H. Kwon M. H. (2016). The effects of leaders’ nonverbal communication on the emotional responses and loyalty of participants in community sports. Korean J. Soc. Phys. Educ. 63, 333–344. doi: 10.51979/KSSLS.2016.02.63.333

[ref9001] Rosenberg N. (Ed.). (2009). Studies on science and the innovation process: Selected works of Nathan Rosenberg. Singapore: World Scientific Publishing. doi: 10.1142/7183

[ref30] Rouiller J. Z. Goldstein I. L. (1993). The relationship between organizational transfer climate and positive transfer of training. Hum. Resour. Dev. Q. 4, 377–399. doi: 10.1002/hrdq.3920040408

[ref31] Schwarz N. Clore G. L. (1983). Mood, misattribution, and judgments of well-being: informative and directive functions of affective states. J. Pers. Soc. Psychol. 45, 513–523. doi: 10.1037/0022-3514.45.3.513

[ref32] Seo J. J. Lee H. J. Park H. R. Lee T. G. (2007). Instructional behavior and interaction of professional golf instructors with learners. Korean J. Sports Educ. 14, 55–69. Available at: https://www.dbpia.co.kr/journal/articleDetail?nodeId=NODE06668408

[ref33] Seol S. H. Kim Y. H. (2010). The impact of service quality on customer satisfaction and revisit intention in screen golf facilities. Korean J. Soc. Sports 41, 255–263. doi: 10.51979/KSSLS.2010.08.41.255

[ref34] Shin J. T. Lee H. W. (2017). The learning and performance effects of golf putting task on enhanced success expectation probability. Korean Soc. Sport Psychol. 28, 83–90. doi: 10.14385/KSSP.28.3.83

[ref35] Won Y. I. Kim S. H. Jung J. H. (2019). The effects of achievement goal orientation on emotional responses and exercise continuation intention among leisure sports participants: focused on golf participants. J. Tour. Leis. Stud. 31, 67–86. doi: 10.31336/JTLR.2019.2.31.2.67

[ref36] Youn S. U. (2021). Tectonic fluctuations from the MZ generation's Golin... Screen golf and golf wear. The JoongAng Ilbo. Available online at: https://news.joins.com/article/24075392 (Accessed June 10, 2025).

